# An improved sample container cup and its effect on NIRS of tobacco smoke for quality stability estimation

**DOI:** 10.1016/j.mex.2023.102478

**Published:** 2023-11-19

**Authors:** Juan Huo, Huaiqi Li, Changtong Lu

**Affiliations:** a Zhengzhou University, Henan, China; b China Tobacco Henan Industrial Co., Ltd, Zhengzhou 450000, China

**Keywords:** Quality control, Near infrared spectroscopy, Tobacco smoke, Smoking machine, Product stability, Chemometrics, Tobacco Smoke NIR for Product stability Evaluation of Cigarettes with new Self-made Container Cup

## Abstract

This paper describes a design of an improved self-made Bruker NIR cup and analyzes the effect of the equipment modification to fit the Cambridge filter pad, which enhances experimental efficiency and reduces operational complexity. A self-made NIR cup based on the classical NIR cup is designed to speed up the operation process and reduce the experiment's time cost. To estimate the effect of this equipment modification, the NIR spectra from the classical sample cup and the new self-made cup are compared and analyzed. Furthermore, the quality evaluation results from NIR data of the two cups are also compared according to a distance metric chemometrics method, which shows quality analytical values between these two cups are approaching each other while the experiment efficiency is improved.

• This paper introduces a newdesign of a self-made container cup improved from the Bruker's traditional sample container cup to better fit the filter pad and improve the experiment efficiency and convenience.
• This paper also analyzes the effect of this container cup change by comparing the NIR spectra before and after modification.

## Graphical abstract

**Figure:**
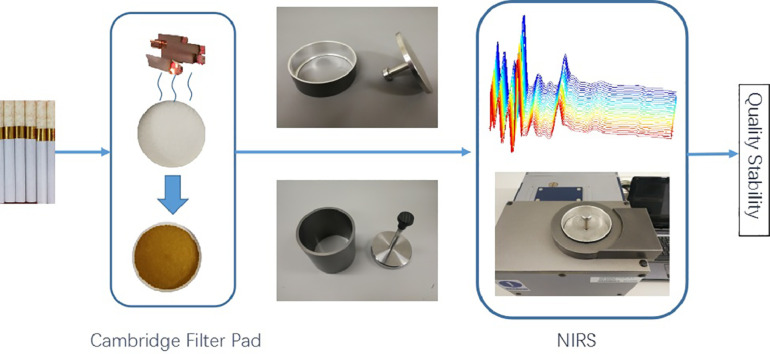

Specifications Table

**Table:** 

Subject Area:	Agricultural and Biological Sciences
More specific subject area:	Chemometrics for tobacco quality control
Method name:	Tobacco Smoke NIR for Product stability Evaluation of Cigarettes with new Self-made Container Cup
Name and reference of original method:	[1] D. L. C, M. S, and C. E, “Rapid Near Infrared Reflectance Analysis of Mainstream Smoke Collected on Cambridge Filter Pads,” Beitrage Zar Tabckforschung International, vol. 16, pp. 171–184, 1995 1995. [2] J. Huo, Y. Ma, C. Lu, L. Chenggang, D. Kun, and L. Huaiqi, “Mahalanobis distance based similarity regression learning of NIRS for quality assurance of tobacco product with different variable selection methods,” Spectrochimica Acta - Part A: Molecular and Biomolecular Spectroscopy, vol. 251, 2021.
Resource availability:	Related R code is available in: https://github.com/JxxxHuo/smokeNIRmah

## Background

The tobacco industry is profoundly influenced by tobacco's smoke and taste as they directly impact the consumer's experience and evaluation of tobacco products. Each tobacco brand possesses unique smoke and taste characteristics. Moreover, tobacco smoke is closely associated with various human health risks, necessitating the maintenance of its characteristics in adherence to specific industrial standards to ensure stability. Traditionally, tobacco smoke estimation has relied on human investigators' smoking experience in tobacco companies, which can be easily influenced by subjective factors. In our previous paper, a method to measure the cigarettes’ periodical product stability was introduced [1]. While numerous methods for tobacco quality control and chemical stability exist, many of them focus on tobacco leaves due to their ease of collection and measurement compared to tobacco smoke [2], [3], [4], [5]. However, it is essential to recognize that the chemical constituents of tobacco leaves and tobacco smoke significantly differ from each other. For instance, tar, a vital chemical component contributing to tobacco taste, is generated through the combustion of tobacco leaves and exists only in tobacco smoke not in tobacco leaves. Capturing and quantitatively analyzing the chemicals in tobacco smoke prove challenging, requiring the use of specialized and expensive instruments designed to generate tobacco smoke NIR accurately [6], [7], [8]. Regarding NIR analysis of tobacco smoke, most experiments have utilized the Cambridge filter pad to collect mainstream smoke particles from the rotary smoking machine, offering a relatively straightforward and cost-effective approach [6,[9], [10], [11], [12], [13]]. Filter pad is also a widely used accessory to store and capture small particles for spectroscopic scan [11,14,15]. However, this process necessitates the modification of optic instrument to fit the pad size or requiring a solvent to extract the smoke particles from the Cambridge filter pad before analyzing their chemical composition [11]. Although the Bruker company has many different kinds of sampling accessories, new design is still needed especially for NIR experiment with special procedures. As an alternative, the research conducted by Parrish et al. employed specialized single-port equipment to capture the smoke puffs and directly NIR scan the smoke with NIR [10]. Nonetheless, this method proves expensive, complex, and impractical for handling a large number of samples. Thus, in this paper, we propose an improvement to the existing equipment based on a method for estimating the product stability of monthly cigarette samples. We have designed a new, self-made Bruker NIR cup to fit the size of the Cambridge filter pad. This modified container cup significantly enhances experimental efficiency and reduces operational complexity. Our method enables the direct determination of product stability using NIRS from the Cambridge filter pad without the need for a solvent. We also conducted an analysis of the effects of equipment modification on test results. The findings demonstrate that while the sample cup modification may slightly shift the NIRs, it has little impact on the calculation of product stability since this stability is determined based on the dissimilarity between the NIRs.

## Materials and equipment

(1) RM20H rotary smoking machine from Borgwaldt kc Company (92 mm diameter Cambridge filter pad)
(2) MATRIX-I FT-NIR spectrometer from Bruker Company (integrating sphere, diffuse reflectance technique)
(3) Bruker standard NIR cup (outer diameter 97 mm; inner diameter 88 mm; transparent quartz glass bottom)
(4) Self-made NIR cup (outer diameter 97 mm; inner top diameter 92 mm; transparent quartz glass bottom; self-made stamper)
(5) Bruker OPUS 6.5 software; TQ Analyst software; Matlab software

### *Method details

**Fig. 1:**
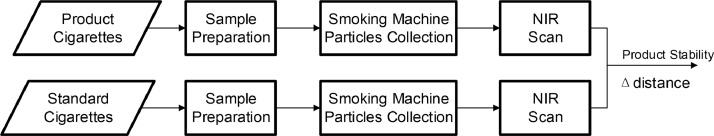
General experiment procedure.

The whole experiment procedure is shown in Fig. 1. The quality is determined by the similarity level between the product samples and the standard samples, which is calculated from the distance metric between smoke NIRS.

1. *Sample preparation*
  (1) Product cigarettes

Every 10 or 15 days, six packs of cigarettes were randomly selected as a batch from the factory products for testing purposes, in both the early and later months. These sample packs were then sealed in plastic bags and stored in a refrigerator at a temperature of −18 °C until the time of testing. The samples can be kept in the refrigerator for a maximum of two years.

  (2) Standard cigarettes

In addition to the product cigarette packs, more than 10 batches of samples, in total, were specifically prepared for each brand as industrial standard samples. Chemical components such as nicotine, tar, and carbon monoxide (CO) in these standard samples were subjected to strict testing. The taste and flavor of these standard samples were examined by professional investigators to confirm the product's sensory characteristics. After thorough preparation, the standard samples were kept in the refrigerator at a temperature of −18 °C.

2. *Smoking machine particles collection*

**Fig. 2:**
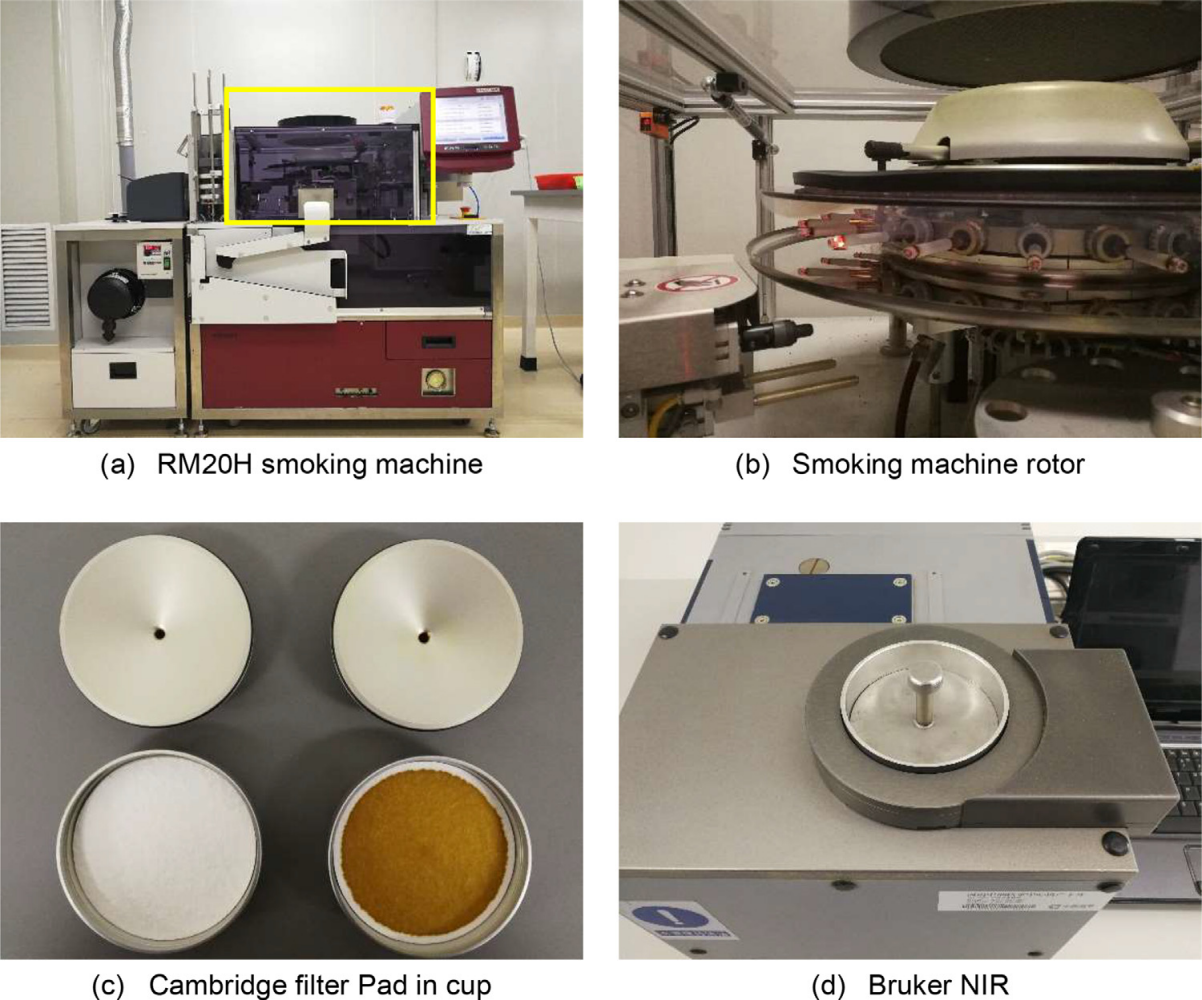
Equipment and procedure of tobacco smoke NIR.

After the required batches of cigarette samples were collected, the samples were taken out from the refrigerator and unpacked. Subsequently, the cigarettes were placed into a conditioned dry box with a constant room temperature of (22 ± 1) °C and a moisture level of (60 ± 3)% for at least 48 h, following the GB/T16447-2004/ISO 3402:1999 standards. Once sufficient balance was achieved, 4080 cigarettes with a full and normal appearance were carefully selected and inserted into the RM20H rotary smoking machine, as shown in Fig. 2(a)(b), to collect the mainstream smoke. To capture the total smoke particles from every 20 cigarettes, a Cambridge filter Pad was utilized, with 24 pads being used for each batch of cigarettes. The smoking rotor in Fig. 2(a) is located in the RM20H smoking machine. All operations were conducted in accordance with the international standards GB/T 19,609–2004 and followed the instructions in the manual of Borgwaldt KC company. After the smoke particles were collected, the Cambridge filter pads were promptly scanned.

3. *NIR scan and new container cup*

The Cambridge filter pads were scanned using a MATRIX-I NIR spectrometer from Bruker company, equipped with an integrating sphere. The machine was preheated for 1 h before the scan commenced. Scanning of these pads was conducted with the full wavenumber range from 4000cm-1 to 12500cm-1, at a resolution of 8cm-1, with each sample being scanned 64 times. The final recorded NIR for each pad is the average of all 64 scans.

The experiment was conducted in an environment with room temperature ranging from 15 °C to 35 °C and moisture levels between 20 % and 70 %. The room temperature variation was kept within 2 °C and monitored every hour, ensured by a remote air conditioner. Additionally, the experiment location was situated far away from any vibration sources, such as transportation centers, motors, wind gaps, or electrical air conditioners.

As each batch of samples has more than 6 pads of smoke particles collected, these Cambridge filter pads were divided into two groups. Half of them were NIR scanned in the old Bruker NIR cup while the other half were tested in the new self-made NIR cup.

(1) Old Bruker NIR cup

**Fig. 3:**
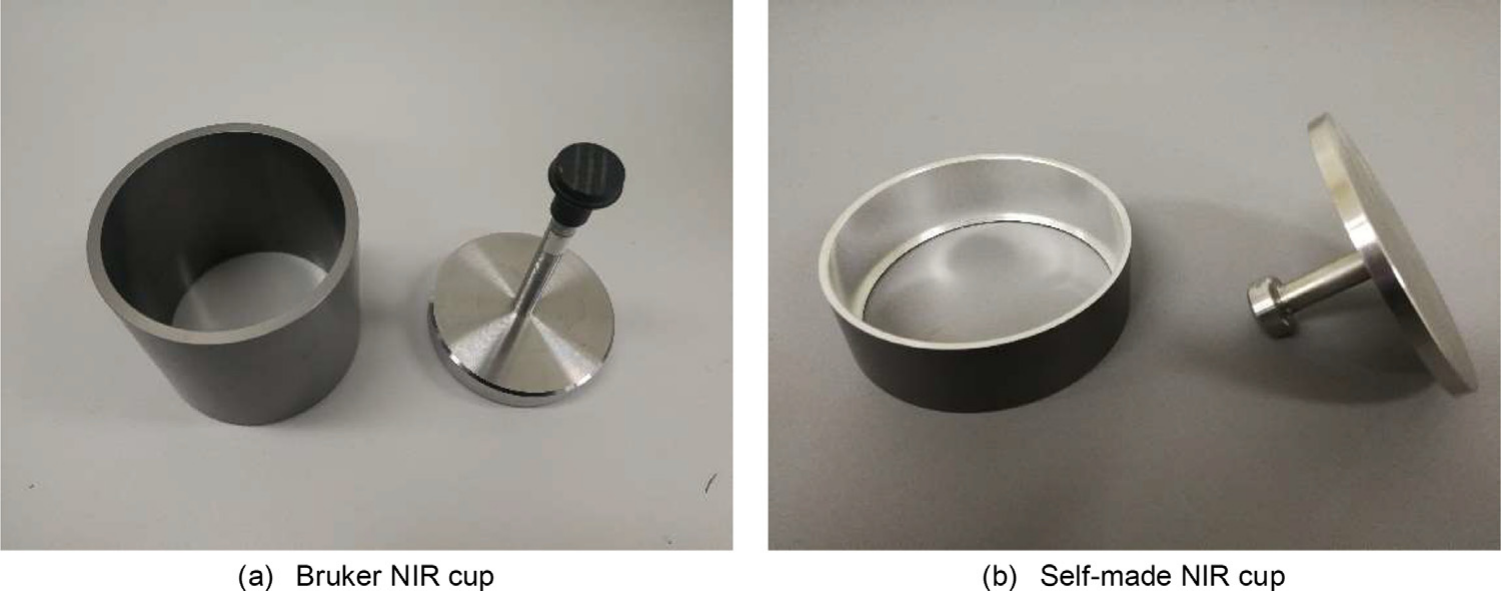
The classical container cup for MATRIX-I Bruker and our new self-made NIR cup.

The pads were typically placed into a classical Bruker NIR cup, as shown in Fig. 3(a), which has a deep, round 88 mm diameter quartz glass bottom, whereas the Cambridge filter pad diameter is 92 mm. To accommodate the pad within the smaller NIR cup bottom of Fig. 3(a), a careful process of cutting the 2 mm wide edge of the Cambridge filter pad was undertaken. This step ensures that the pad's edge remains smooth and can be attached closely to the inner wall of the cup, preventing any potential optic leaks.

Given the tall and deep design of the Bruker cup, an additional time-consuming step involves pressing the pad carefully onto the bottom of the cup. The removed edge of the pad was positioned at the top of the NIR cup, with the side containing the freshly collected smoke particles facing downward. Subsequently, the pad was slowly pushed to the bottom of the cup using a stamper. Throughout the NIR scan, the stamper remained in place above the pad.

If the pad failed to cover the cup bottom completely or if there was any indication of optic leakage, it was deemed unsuitable for further use and subsequently abandoned. After each scan was completed, the quartz glass bottom was cleaned with a soft cotton material.

(2) New self-made NIR cup

Since there is a need to test hundreds of samples within a short time, it is essential to reduce the sample preparation time. As a result, we have designed a new NIR cup, shown in Fig. 3(b), that is only 50 mm tall, significantly shorter than the old cup. While the outer diameter of the new cup remains the same as the old one at 97 mm, the inner diameter of the new cup is 92 mm at the top and 88 mm at the bottom. The bottom of the cup consists of transparent quartz glass that is 1 mm high. Both the new cup top and its stamper have a diameter of 92 mm, matching the Cambridge filter pad's diameter exactly.

Thanks to this design, there is no need to cut off the pad's edge with scissors, and the Cambridge filter pad can be easily pushed to the bottom of the cup. Moreover, the new stamper, being heavier, ensures that the pad border adheres closely to the inner cup bottom border. The diameter of the glass cup bottom in the last 1 mm depth is smaller than the metal border, preventing the smoke particle pad from sticking to the quartz glass bottom while remaining close enough for correct scanning. This design makes the bottom easy to clean and free from remaining particle pollution.

Most importantly, the above design of the new cup significantly speeds up the NIR experiment. The faster preprocessing of the Cambridge filter pad before the NIR scan reduces the air contact time and the potential pollution.

4. Similarity based product stability estimation

After collecting both the products and standard NIRS, they were first preprocessed using OPUS analysis software to reduce baseline drift and noise before calculating the product stability metric. The NIRS preprocess involved three steps: Savitzky-Golay smoothing, multiplicative scatter correction (MSC), and first-order derivatives with second-order polynomial approximation.

In this research, product stability is determined based on the similarity level between the NIR of the products and the standard NIR. Using similarity estimation for quality control offers two advantages: firstly, as there are thousands of chemical components in tobacco combustion products, concentration analysis can be challenging and not mandatory; secondly, the similarity calculation can mitigate the effects of empty Cambridge filter pads.

**Fig. 4:**
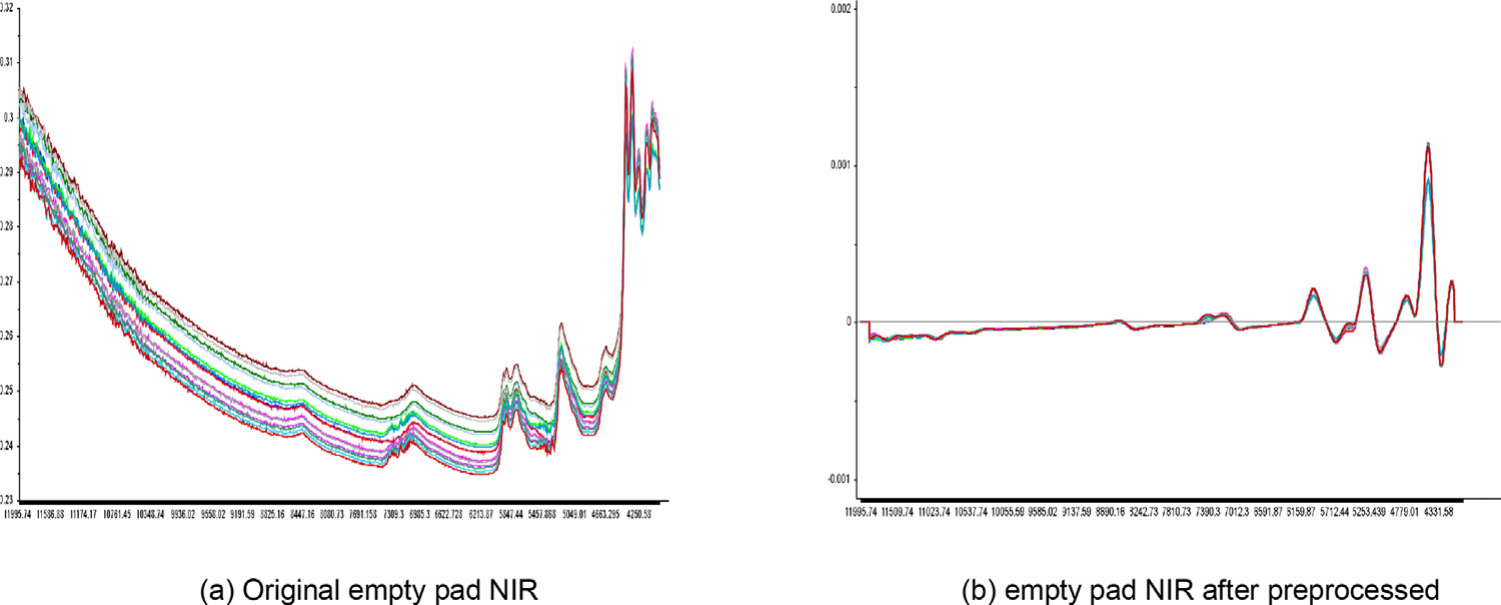
NIRS of empty Cambridge filter pad.

Fig. 4(a) shows 12 NIR spectra of empty Cambridge filter pads. After applying the same preprocess steps as the tobacco NIR, the first-order derivative of the empty pad NIRS (Fig. 4(b)) exhibits almost identical shapes with only slight differences in certain spectral regions when compared to the amplitude of the tobacco NIR. Thus, the impact of empty Cambridge filter pads on similarity calculation is limited, and the quality similarity level can be directly inferred from NIRS of pads.

Various algorithms can represent the similarity level, such as similarity match value, distance match value, spectral correlation, and information divergence .among others [16,17]. For the latter experiment, Mahalanobis distance is chosen as the similarity metric, a widelyused method for NIR discrimination and classification [16,18].

## Experiment data comparison

**Fig. 5:**
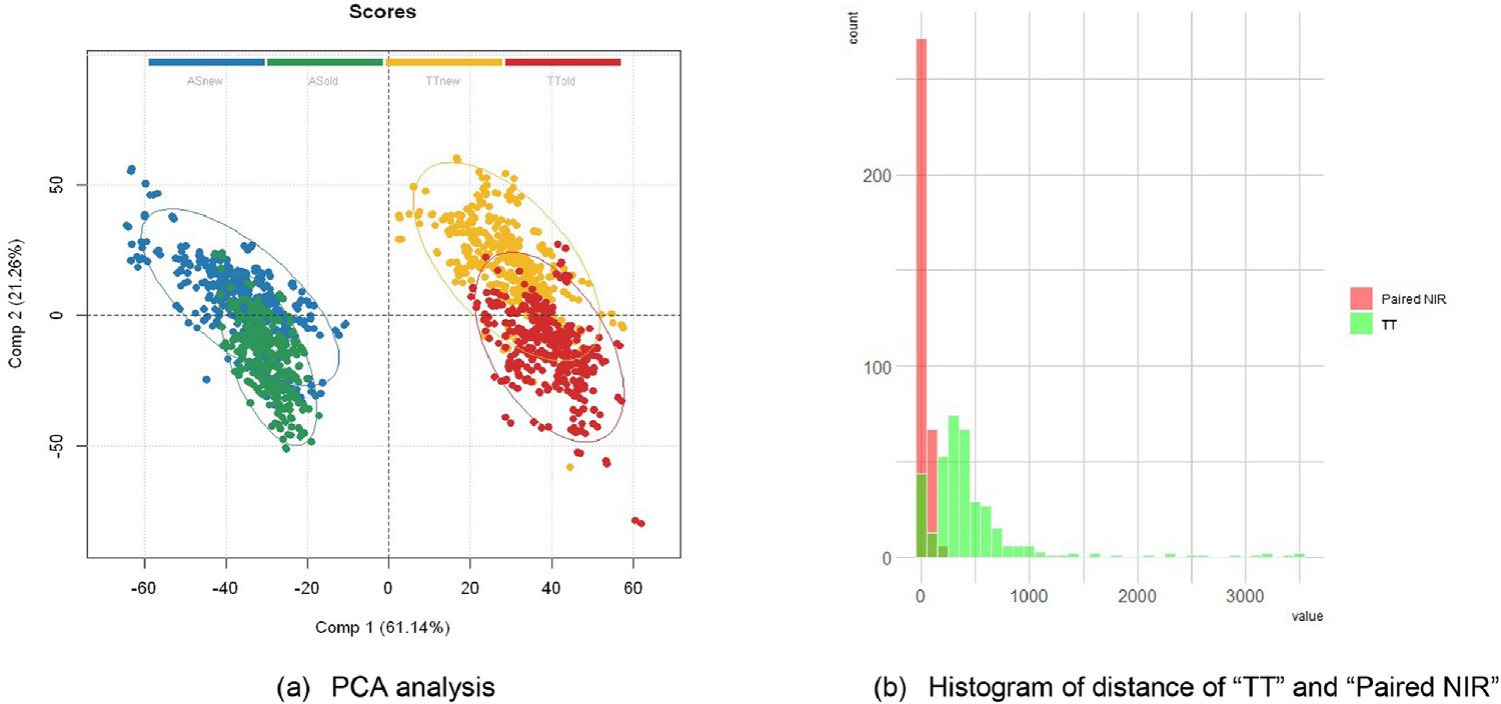
(a) PCA of “AA” and “TT” brands NIRS. (b) Histogram of the similarity distance of different comparison groups of Fig. 6(b).

NIRS samples were prepared over a span of two years, comprising 62 batches of the “TT” brand and 66 batches of the “AA” brand. The scanning of these samples followed the procedure shown in Fig. 1, and the spectra were collected using the same spectrometer over a few days. A total of 772 NIR samples were generated from examining the cigarette samples using this method. Among these, there were 44 standard NIRS from the “TT” brand and 55 standard NIRS from the “AA” brand. Principal Component Analysis (PCA) was initially applied to these spectra, and the results are depicted in Fig. 5(a). The principal components of both the old and new cup NIRS were visualized, showing clear separation between the “AA” and “TT” brands, irrespective of whether the old or new NIR cup type was used. However, within each brand, the NIRS clusters from the old and new cups partially overlapped.

Fig. 5 shows the chemometrics analysis results of NIR. In Fig. 5(a), the PCA analysis displays the distribution of all NIRS from both “TT” and “AA” brands. The blue dots represent the “AA” NIRS scanned in the old Bruker cup, while the green points are the “AA” NIRS scanned in the new self-made cup. The yellow dots correspond to the “TT” NIRS scanned in the old Bruker NIR cup, and the red points represent the “TT” NIRS scanned in the new self-made NIR cup. Fig. 5(b) shows the histogram of the Mahalanobis similarity distance of “Paired NIR” within either “AA” or “TT” brand between old and new cup as red bar, and distance between “TT” sample and standard “TT” NIR.

To further observe the variation of similarity level along the timeline, this experiment utilizes the Mahalanobis distance as the distance match value between the averaged NIR of each product batch and the standard NIRS. Since there are multiple measured NIR spectra for the standard samples due to statistical reasons, using a metric capable of estimating the distance between an observation and a distribution is necessary. The Mahalanobis distance algorithm accomplishes this by multidimensionally generalizing the number of standard deviations from an observation to the mean of a set of observations., which can be expressed as:

$$D=\sqrt{\left(x-\mu \right)C^{-1}{\left(x-\mu \right)}^{T}}$$

Where x is the vector of the products NIR and μ is the mean of the standard NIR set and C here represents the covariance of standard NIR sets as reference. In the following discussion, the referenced standard sets are “TT” brand's. The distance D is generally symmetric for “AA” and “TT” no matter which brand is considered as the standard reference.

**Fig. 6:**
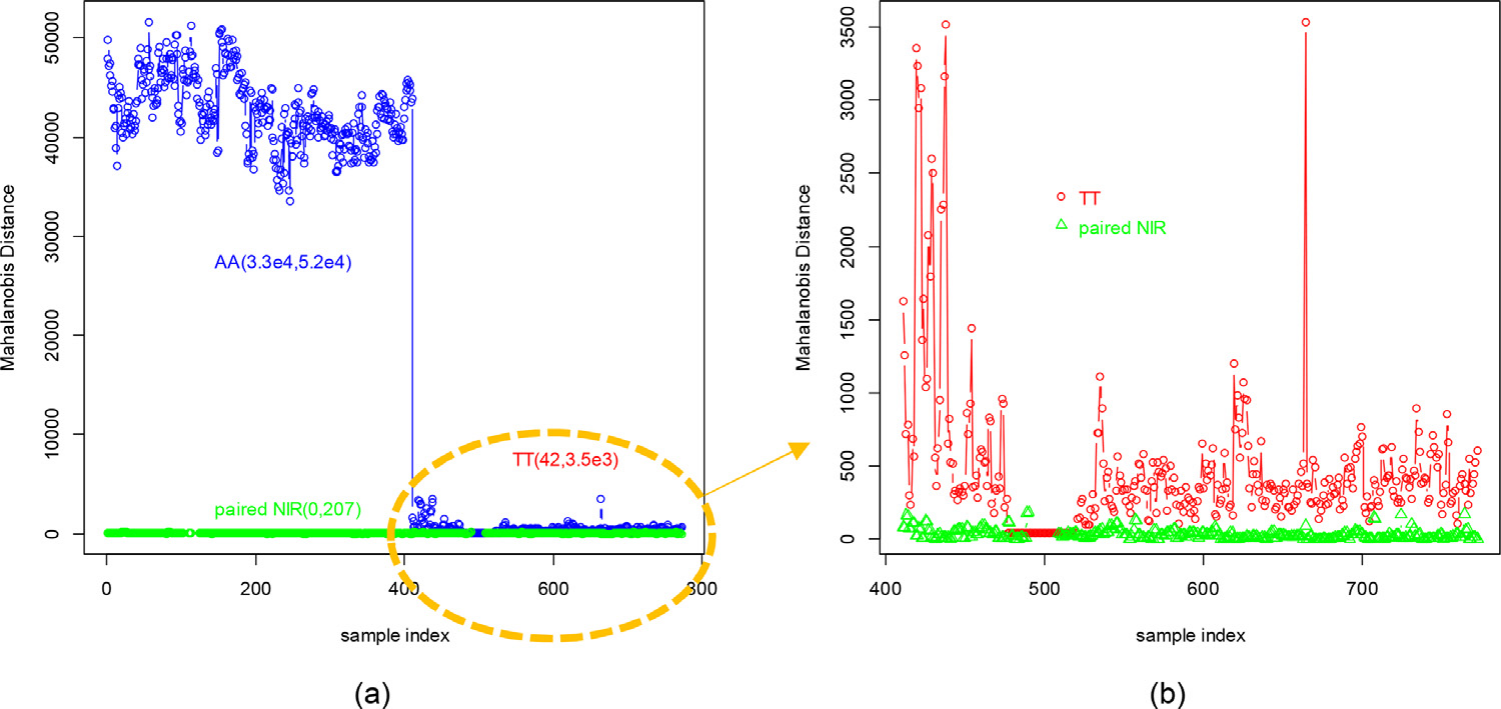
The Mahalanobis distance between NIRS of all 772 NIR samples. The blue line represents the Mahalanobis distance between the “AA” sample NIRS and the “TT” standard NIRS, while the red line represents the distance between the “TT” sample NIRS and the “TT” standard NIRS. Additionally, the green line represents the Mahalanobis distance between the paired NIRS scanned by the Bruker cup and our new self-made new cup within either “AA” in (a) or “TT” in (b).

Fig. 6 is utilized to represent the intra and inter-brands similarity differences. The Mahalanobis distance is essentially inverse to the similarity level between the NIRS. For the inter-brands similarity difference, the “AA” NIR samples, being quite different from TT”.The blue line of “AA” NIR exhibits a high Mahalanobis distance value ranging from 3.3 × 10^4^ to 5.2 × 10^4^. In contrast, the “TT” NIR samples have a much smaller range from 42 to 3.5 × 10^3^. Concerning the intra-brands similarity difference, the green line represents the paired NIRS of the same cigarette sample source, scanned from the old Bruker and new self-made NIR cup separately. The paired NIR ranges from 0.81 to 218. The Mahalanobis distance, shown in Fig. 6(b), is then statistically estimated as a histogram in Fig. 5(b), indicating that the “TT” distance distribution center is significantly higher than the paired NIR distance. The difference between the paired NIR is small and stable, confirming that the new self-made cup yields consistent NIR test results, similar to the old NIR cup.

**Fig. 7:**
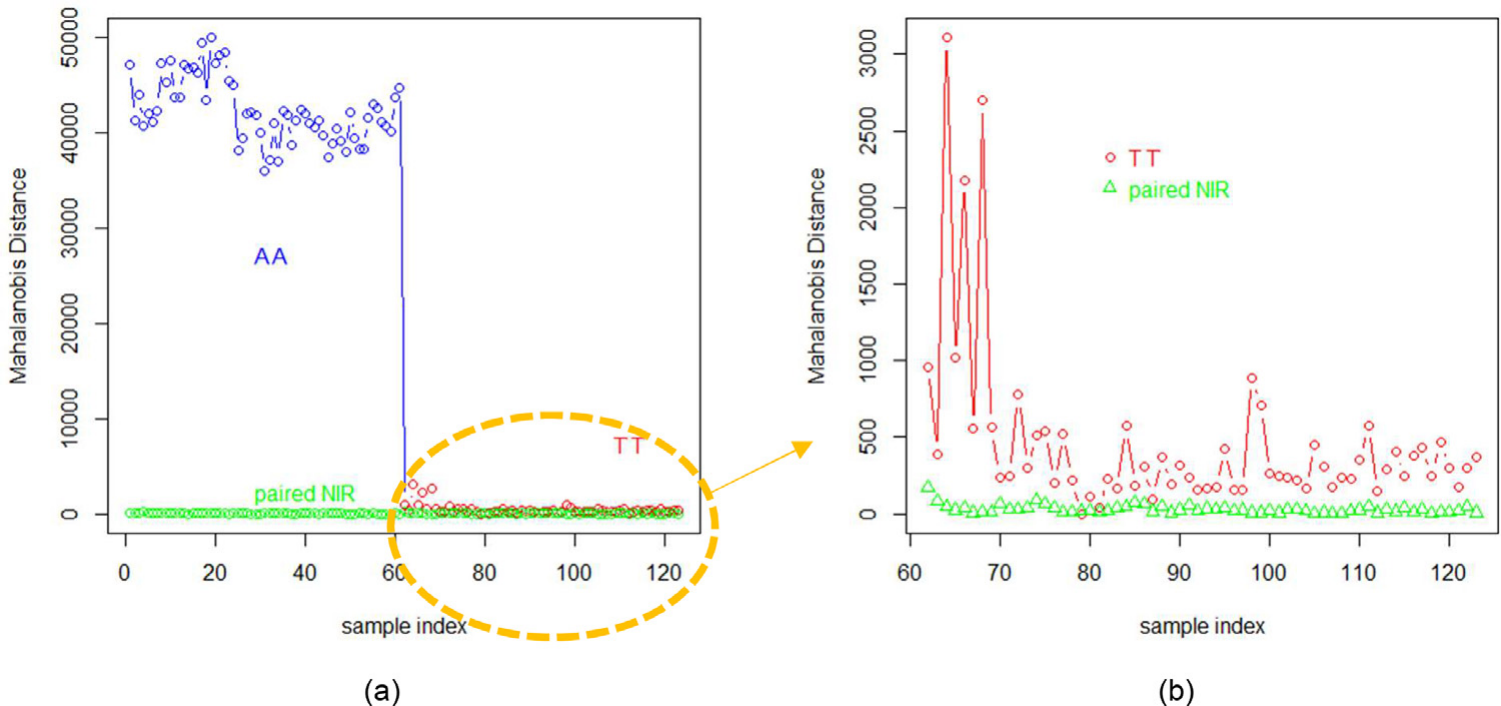
The averaged Mahalanobis distance of the 128 batches.

Finally, the NIRS of each batch were averaged to observe the monthly quality change, reducing anomalies caused by the environment and Cambridge filter pads through the averaging process. Fig. 7(b) indicates that the quality variation within the “TT” brands is very small, except for the initial two months' samples, which is consistent with the human investigators' report, as the initial two months' samples had been stored much longer than the later standard samples. Meanwhile, the original four steps filter pad preparation process becomes three steps as the time-cost pad size adjustment is eliminated. The low depth cup is much easier to be cleaned after a pad is scanned, which makes the scan efficiency double.

## Conclusion

This paper introduces a new self-made container cup for Bruker Matrix-I NIR instrument to improve the lab NIR efficiency. This paper aimed to improve the method of tobacco smoke measurement for product stability estimation by modifying the sample container cup for easier operation and measurement. Product stability was estimated from the similarity level-based distance match value between the NIRS measured from the Cambridge filter pad, which was pushed onto the back of the self-made container cup. Experiment analysis shows that the modification of the self-made NIR container cup in this paper has little effect on stability estimation and the modification can effectively reduce tobacco smoke NIR experiment time cost to speed up the operation process.

## Discussion

The product stability estimation method presented in this paper is generally applicable to most tobacco brands with a single tobacco leaves source. However, for cigarettes made from a mixture of multiple types of tobacco leaves, it is recommended to combine the product stability estimation method described in this paper with other machine learning related algorithms to enhance accuracy, as detailed in our related paper [1].

## Declaration of Competing Interest

The authors declare that they have no known competing financial interests or personal relationships that could have appeared to influence the work reported in this paper.

## Data Availability

Part of the data and code have been shared publicly while the other part is confidential.

## References

[1] Huo J., Ma Y., Lu C., Li C., Duan K., Li H. (2021). Mahalanobis distance based similarity regression learning of NIRS for quality assurance of tobacco product with different variable selection methods. Spectrochim. Acta Part A Mol. Biomol. Spectrosc..

[2] Marcelo M., Soares F., Ardila J., Dias J., Pedó R., Kaiser S. (2019). Fast inline tobacco classification by near-infrared hyperspectral imaging and supporting vector machines-discriminant analysis. Anal. Methods.

[3] Y. Bi, S. Li, L. Zhang, Y. Li, W. He, J. Tie, et al., Quality evaluation of flue-cured tobacco by near infrared spectroscopy and spectral similarity method vol. 215, 2019. 10.1016/j.saa.2019.01.094 30865909

[4] Zhiqiang R., Hongxiang T., null B., Qian C. (2013). Application of principal component analysis in evaluation of cigarette quality and its consistence. Tob. Sci. Technol..

[5] Chen X., Wang W.Y., Liu Y., Xia J.J., Jiang J.X., Li Q.Q. (2013). Quality control of tobacco top flavor by NIR spectral similarity match analysis. Laser Infrared.

[6] Gasparyan H., Mariner D., Wright C., Nicol J., Murphy J., Liu C. (2018). Accurate measurement of main aerosol constituents from heated tobacco products (HTPs): implications for a fundamentally different aerosol. Regul. Toxicol. Pharmacol..

[7] Adamson J., Azzopardi D., Errington G., Dickens C., McAughey J., Gaça M.D. (2011). Assessment of an *in vitro* whole cigarette smoke exposure system: the Borgwaldt RM20S 8-syringe smoking machine. Chem. Cent. J..

[8] Borges-Miranda A., Silva-Mata F.J., Talavera-Bustamante I., Jiménez-Chacón J., Álvarez-Prieto M., Pérez-Martínez C.S. (2021). The role of chemosensory relationships to improve raw materials’ selection for Premium cigar manufacture. Chem. Pap..

[9] Q. Fu, X. Wang, J. Ge, H. Zhang, Y. Du, X. Hou, et al., "Prediction of tar, nicotine and CO release amount in mainstream smoke of cigarette by near infrared spectroscopy," Infrared Technology, vol. 36, pp. 249–254, 2014 2014.

[10] Parrish M.E., Lyons-Hart J.L., Shafer K.H. (2001). Puff-by-puff and intrapuff analysis of cigarette smoke using infrared spectroscopy. Vib. Spectrosc..

[11] Di Luzio C., Morzilli S., Cardinale E. (1995). Rapid near infrared reflectance analysis (NIRA) of mainstream smoke collected on Cambridge filter pads. Beitraege Tabakforsch. Int..

[12] Clayton P., Cunningham A., Van Heemst J. (2010). Quantification of four tobacco-specific nitrosamines in cigarette filter tips using liquid chromatography-tandem mass spectrometry. Anal. Methods.

[13] Wu W., Ashley D.L., Watson C.H. (2003). Simultaneous determination of five tobacco-specific nitrosamines in mainstream cigarette smoke by isotope dilution liquid chromatography/electrospray ionization tandem mass spectrometry. Anal. Chem..

[14] Roesler C., Stramski D., D'Sa E., Röttgers R., Reynolds R.A. (2018).

[15] Wooten J.B. (2011). Gas-phase radicals in cigarette smoke: a re-evaluation of the steady-state model and the Cambridge filter pad. Mini Rev. Org. Chem..

[16] TQ Analyst software user guide. Madison (WI): Thermo Fisher Scientific, 2011.AU: Please provide complete details in Refs. [3,9,16].

[17] Li W.-l., Han H.-f., Zhang L., Zhang Y., Qu H.-b. (2016). Manufacturer identification and storage time determination of “Dong'e Ejiao” using near infrared spectroscopy and chemometrics. J. Zhejiang Univ. Sci. B.

[18] Mishra P., Nordon A., Tschannerl J., Lian G., Redfern S., Marshall S. (2018). Near-infrared hyperspectral imaging for non-destructive classification of commercial tea products. J. Food Eng..

